# Risk of Bleeding Associated With Antidepressants: Impact of Causality Assessment and Competition Bias on Signal Detection

**DOI:** 10.3389/fpsyt.2021.727687

**Published:** 2021-10-21

**Authors:** René Zeiss, Bernhard J. Connemann, Carlos Schönfeldt-Lecuona, Maximilian Gahr

**Affiliations:** Department of Psychiatry and Psychotherapy III, University of Ulm, Ulm, Germany

**Keywords:** antidepressants, serotonin transporter, bleeding risk, pharmacovigilance, competition bias

## Abstract

**Introduction:** It has not yet been possible to demonstrate the well-established increased bleeding risk related to antidepressants (ADs) with methods of pharmacovigilance as disproportionality analysis. As bleeding events related to ADs often occur under comedication with antithrombotics, ADs might not be considered causative of, but merely “linked” with the bleeding event. Therefore, we hypothesized that causality assessment of bleeding events related to ADs and the competitive impact of antithrombotics are factors contributing to the mentioned previous non-findings.

**Methods:** We performed a case/non-case study based on data from VigiBase™ and calculated reporting odds ratios (RORs) for 25 ADs. We used individual case safety reports (ICSRs) that were differently categorized in the database regarding the type of association between drug and event. Furthermore, we investigated the competitive impact of antithrombotics by comparing RORs calculated with and without ICSRs related to antithrombotics.

**Results:** Analysis of ICSRs that were categorized as causally associated with ADs resulted in detection of only 2 signals (citalopram and escitalopram; upper gastrointestinal bleeding). Analysis of ICSRs irrespective of the type of association resulted in detection of 8 signals (regarding bleeding in general, gastrointestinal bleeding and upper gastrointestinal bleeding). In our analysis, consideration of ICSRs associated with antithrombotics as competitive substances did not have significant impact on signal detection.

**Conclusion:** Categorization of the type of association between drug and event may affect quantitative signal detection toward reduced sensitivity. Causality assessment seems to significantly impact signal detection, probably particularly in rare, unknown, or clinically unremarkable adverse drug reactions. ADs appear to increase the bleeding risk considerably, even independent of antithrombotic comedication.

## Introduction

Antidepressants (ADs) are frequently used in the treatment of depression, anxiety disorders, and several other mental disorders (1–4). In addition, the use of antidepressants has also increased in the elderly population and in patients suffering from severe somatic illnesses (e.g., acute coronary syndrome) (5). However, in the last years, several studies suggested an increased bleeding risk related to ADs, particularly agents that inhibit the reuptake of serotonin, and in regard to particular risk groups, e.g., patients taking antithrombotic drugs (6, 7). There are studies suggesting an increased risk for brain haemorrhages (8, 9), peri- and postoperative bleeding (10), and gastrointestinal haemorrhages (11), especially of the upper gastrointestinal tract (12–14) related to ADs. Until now it was not possible to demonstrate the otherwise well-established increased risk of bleeding related to ADs with disproportionality analysis using data of pharmacovigilance databases (15, 16). We hypothesize that insufficient consideration of comedication and reporter-related factors that affect causality assessment and documentation of adverse drug reaction (ADR) reports in the database may be relevant for these non-findings. Furthermore, in the analysis of data from pharmacovigilance databases, such as the European EudraVigilance, the FDA Adverse Event Reporting System (FAERS) or the adverse drug reaction (ADR) database of the WHO, VigiBase^*TM*^, several biases need to be taken into account, for example, the notoriety bias (17), underreporting (18, 19), and the competition bias (20, 21). The competition bias describes the phenomenon that a substance or an event that is frequently associated with the event of interest may (competitively) “mask” a substance-related signal (20, 22). Moreover, the evaluation of the type of the association between drug and event by the person who reports the respective ADR (=reporter) may affect the results of signal detection, too. Drug/event associations can be reported to the ADR database as “concomitant” (not suspected as causally linked, but merely “associated”) or “suspected/interacting” (suspected as causally linked). According to the International Council for Harmonization (ICH) guidelines, the characterization of the drug's causality for the event is made by the primary reporter. For instance, a bleeding event occurring under a medication including a selective serotonin reuptake inhibitor (SSRI) and a non-steroidal anti-inflammatory drug (NSAID) could be reported as an ADR caused by the NSAID alone, if the reporting person is unfamiliar with the bleeding risk related to SSRI. As a consequence, the association with the SSRI is documented as “concomitant” in the database, and a possible causal relation is not adequately considered. The impact of the categorization as “concomitant” or “suspected/interacting” on the results of disproportionality analysis regarding the bleeding risk of ADs has not yet been studied. This consideration might be of particular importance as disproportionality analysis has not yet been able to demonstrate the bleeding risk related to ADs. Therefore, in the present paper, we (i) evaluated the impact of the categorization of drug/event (ADs/haemorrhages) reports as causally linked (category: “suspected/interacting”) versus all types of association/irrespective of the type of association (category: “suspected/interacting/concomitant”) on signal detection, (ii) evaluated the risk of different types of bleeding events (haemorrhages, in general, gastrointestinal bleeding and upper gastrointestinal bleeding) related to antidepressants, and (iii) analyzed the impact of the competition bias by evaluating the role of antithrombotics as competitive substances.

## Methods

### Database and Database Query

The collection of data was done by a data search query at VigiBase™ (date of database query: December 10, 2018) using terms of the Medical Dictionary for Regulatory Activities (MedDRA). VigiBase™ contains over 20 million reports of individual case safety reports (ICSRs) (23). The search terms used were the standardized MedDRA Queries (SMQs) “Haemorrhage” (narrow scope), “Gastrointestinal haemorrhage,” and the preferred term (PT) “Upper gastrointestinal haemorrhage.” The MedDRA Version used was 21.1. The number of ICSRs related to the following ADs associated with each of the abovementioned conditions were extracted: agomelatine, amitriptyline, bupropion, citalopram, clomipramine, doxepin, duloxetine, escitalopram, fluoxetine, fluvoxamine, hypericum perforatum, imipramine, maprotiline, milnacipran, mirtazapine, moclobemide, nortriptyline, paroxetine, reboxetine, sertraline, tianeptine, tranylcypromine, trazodone, trimipramine, and venlafaxine.

### Categorization of Individual Case Safety Reports

In the first analysis, the categorization chosen for the database query was “suspected/interacting” and, for the second analysis, “suspected/interacting/concomitant.” The categorization of a drug/event report as “suspected,” respectively, “interacting” (meaning that a causal relation between the drug and event is assumed) or “concomitant” (meaning that only an association between drug and event in the database is present, e.g., the drug is part of a polypharmacy and administered before or during the occurrence of the event of interest) is made by the primary reporter of the ICSR. All drugs that are considered as “interacting” are also considered to be “suspected” drugs in the database (24). Drugs that are considered as neither “suspected” nor “interacting” are categorized as “concomitant.” The category used in the second analysis was “suspected/interacting/concomitant” and comprises all ICSRs irrespective of the available information regarding the type of the association between drug and event.

### Statistical Analysis

We performed a case/non-case study and calculated the reporting odds ratios (ROR) and the 95% confidence intervals (CI) for disproportionality analysis. For this type of study, all reports associated with the respective substance and the ADR of interest are defined as “cases” and all other reports in the database as “non-cases.” If an ADR related to a certain substance is reported more frequently in comparison with all other substances in the database, this is referred to as a signal. In this study, a signal is defined as a ROR >1 with a lower limit of the 95% CI >1. First, the RORs were calculated for both datasets (“suspected/interacting” and “suspected/interacting/concomitant”); in the second dataset (“suspected/interacting/concomitant”), the competitive impact of antithrombotics was evaluated as described in the following paragraph (2.4). In a previous analysis where only ICSRS categorized as “suspected/interacting” were analyzed, the effects of the competition bias, i.e., the comparison of RORs calculated with vs. without inclusion of ICSRs associated with antithrombotics, were found to be negligible [see Zeiss et al. (25) for details]. Data analysis was performed with Microsoft® Excel Version 16.16.8 and RStudio Version 1.2.5019.

### Competition Bias

For the dataset “suspected/interacting/concomitant,” we made the calculation without considering the competition bias by using the entire data available in the database. For consideration of the competition bias, we calculated the RORs after removing all reports associated with a substance of the group “antithrombotic agents” [Anatomical Therapeutic Chemical (ATC) Classification System code B01]. These substances were chosen because haemorrhages are considered as a so-called type A reaction related to antithrombotic agents, meaning that the ADR is a consequence of the pharmacological effect and therefore very common (22).

## Results

At the time of the database query, there were 18,709,028 ICSRs in VigiBase^*TM*^, 990,119 of them were associated with the SMQ “Haemorrhage,” 257,500 with the SMQ “Gastrointestinal Haemorrhage,” and 10,908 with the PT “Upper gastrointestinal haemorrhage.” In the following, we will present the signals found for each of the investigated SMQs, respectively, PT, first in the analysis of ICSRs that were categorized as “suspected/interacting,” followed by “suspected/interacting/concomitant.” For the latter, signals detected after removal of ICSRs related to antithrombotics are additionally presented. The comparison between the ROR values for “suspected/interacting” vs. “suspected/interacting/concomitant” is presented in Supplementary Tables S1–S3.

### Standardized MedDRA Queries “Haemorrhage”

In the analysis of ICSRs categorized as “suspected/interacting” concerning the SMQ “haemorrhage” (ICSRs related to antithrombotics included; no consideration of the competition bias), no signal was found; using ICSRs categorized as “suspected/interacting/concomitant,” eight signals were detected: amitriptyline [ROR: 1.09 (95% CI, 1.06–1.12)], citalopram [ROR: 1.41 (1.38–1.45)], escitalopram (ROR: 1.35 (1.32–1.39), fluoxetine [ROR: 1.03 (1.01–1.05)], hypericum perforatum [ROR: 1.26 (1.10–1.45)], paroxetine [ROR: 1.05 (1.02–1.07)], sertraline [ROR: 1.22 (1.20–1.25)], and trazodone [ROR: 1.28 (1.25–1.32)]. After removal of ICSRs related to antithrombotics (i.e., consideration of the competition bias), nine signals were found (using ICSRs categorized as “suspected/interacting/concomitant”): Two new signals related to venlafaxine [ROR: 1.06 (1.02–1.09) and duloxetine [ROR: 1.07 (1.04–1.11)] were detected, while the signal related to paroxetine became non-significant [ROR 1.02 (0.99–1.05)]; the other signals persisted. Regarding the ROR values including the 95% CI for each substance for SMQ “Haemorrhage,” see Table 1.

**Table 1:** Reporting odds ratios and 95% CIs related to antidepressants and the SMQ “haemorrhage” with and without consideration of the competition bias.

**Substance**	**ROR without consideration of the competition bias**	**ROR in consideration of the competition bias**
Agomelatine	0.54 (0.45–0.65)	0.59 (0.47–0.73)
Amitriptyline	1.09 (1.06–1.12)	1.17 (1.13–1.21)
Bupropion	0,79 (0.77–0.81)	0.93 (0.90–0.96)
Citalopram	1.41 (1.38–1.45)	1.33 (1.29–1.37)
Clomipramine	0.59 (0.54–0.65)	0.67 (0.60–0.74)
Doxepin	0.99 (0.92–1.06)	1.04 (0.95–1.13)
Duloxetine	0.97 (0.95–1.00)	1.07 (1.04–1.11)
Escitalopram	1.35 (1.32–1.39)	1.31 (1.27–1.36)
Fluoxetine	1.03 (1.01–1.05)	1.19 (1.16–1.22)
Fluvoxamine	0.62 (0.57–0.68)	0.74 (0.67–0.82)
Hypericum perforatum	1.26 (1.10–1.45)	1.62 (1.40–1.89)
Imipramine	0.80 (0.73–0.87)	0.90 (0.82–1.00)
Maprotiline	0.52 (0.44–0.62)	0.59 (0.49–0.72)
Milnacipran	0.77 (0.68–0.87)	0.94 (0.82–1.08)
Mirtazapine	1.04 (1.00–1.08)	0.82 (0.78–0–86)
Moclobemide	0.37 (0.30–0.45)	0.42 (0.33–0.52)
Nortriptyline	1.06 (1.00–1.12)	1.17 (1.09–1.26)
Paroxetine	1.05 (1.02–1.07)	1.02 (0.99–1.05)
Reboxetine	0.40 (0.31–0.51)	0.54 (0.41–0.70)
Sertraline	1.22 (1.20–1.25)	1.28 (1.24–1.31)
Tianeptine	1.10 (0.95–1.26)	0.49 (0.38–0.65)
Tranylcypromine	0.72 (0.58–0.88)	0.98 (0.79–1.21)
Trazodone	1.28 (1.25–1.32)	1.26 (1.22–1.31)
Trimipramine	0.86 (0.75–0.98)	0.90 (0.76–1.06)
Venlafaxine	0.97 (0.95–1.00)	1.06 (1.02–1.09)

*CI, confidence interval; ROR, reporting odds ratio; SMQ, standardized MedDRA queries*.

### Standardized MedDRA Queries “Gastrointestinal Haemorrhage”

In the analysis of ICSRs categorized as “suspected/interacting” regarding the SMQ “Gastrointestinal Haemorrhage” (ICSRs related to anti-thrombotics included; no consideration of the competition bias), no signals were found. Using ICSRs categorized as “suspected/interacting/concomitant,” eight signals were found: amitriptyline [ROR: 1.43 (95% CI, 1.37–1.50)], citalopram [ROR: 1.67 (1.60–1.74)], doxepin [ROR: 1.28 (1.14–1.43)], escitalopram [ROR: 1.37 (1.30–1.43)], mirtazapine [ROR: 1.41 (1.33–1.49)], paroxetine [ROR: 1.10 (1.05–1.15)], sertraline [ROR: 1.31 (1.26–1.36)], and trazodone [ROR: 1.56 (1.49–1.64)]. After removing reports associated with antithrombotics from the database (i.e., consideration of the competition bias) 10 signals were found, from which four were new (using ICSRs categorized as “suspected/interacting/concomitant”): duloxetine [ROR: 1.13 (1.05–1.20)], fluoxetine [ROR: 1.07 (1.01–1.14)], nortriptyline [ROR: 1.18 (1.02–1.38)], and venlafaxine [ROR: 1.10 (1.03–1.17)]; the previously found signal for mirtazapine [ROR: 1.41 (1.33–1.49) before and 1.10 (1.00–1.21) after removal] and paroxetine [ROR: 1.10 (1.05–1.15) vs. 1.04 (0.98–1.11)] disappeared. For the ROR values including the 95% CI for each substance for the SMQ “Gastrointestinal Haemorrhage,” see Table 2.

**Table 2:** Reporting odds ratios and 95% CIs related to antidepressants and the SMQ “Gastrointestinal Haemorrhage” with and without consideration of the competition bias.

**Substance**	**ROR without consideration of the competition bias**	**ROR in consideration of the competition bias**
Agomelatine	0.42 (0.28–0.62)	0.51 (0.31–0.84)
Amitriptyline	1.43 (1.37–1.50)	1.65 (1.55–1.75)
Bupropion	0.69 (0.65–0.73)	0.81 (0.75–0.88)
Citalopram	1.67 (1.60–1.74)	1.50 (1.41–1.60)
Clomipramine	0.48 (0.40–0.58)	0.57 (0.45–0.73)
Doxepin	1.28 (1.14–1.43)	1.43 (1.23–1.66)
Duloxetine	1.01 (0.96–1.06)	1.13 (1.05–1.20)
Escitalopram	1.37 (1.30–1.43)	1.30 (1.22–1.40)
Fluoxetine	0.96 (0.92–1.00)	1.07 (1.01–1.14)
Fluvoxamine	0.63 (0.53–0.75)	0.79 (0.64–0.97)
Hypericum perforatum	1.02 (0.77–1.36)	0.95 (0.63–1.43)
Imipramine	0.95 (0.82–1.10)	1.10 (0.91–1.33)
Maprotiline	0.45 (0.32–0.63)	0.51 (0.33–0.79)
Milnacipran	0.46 (0.34–0.62)	0.54 (0.37–0.79)
Mirtazapine	1.41 (1.33–1.49)	1.10 (1.00–1.21)
Moclobemide	0.46 (0.33–0.65)	0.55 (0.36–0.84)
Nortriptyline	1.05 (0.93–1.17)	1.18 (1.02–1.38)
Paroxetine	1.10 (1.05–1.15)	1.04 (0.98–1.11)
Reboxetine	0.37 (0.22–0.62)	0.58 (0.34–1.00)
Sertraline	1.31 (1.26–1.36)	1.27 (1.20–1.34)
Tianeptine	1.01 (0.77–1.34)	0.48 (0.26–0.86)
Tranylcypromine	0.27 (0.14–0.52)	0.43 (0.21–0.85)
Trazodone	1.56 (1.49–1.64)	1.41 (1.31–1.52)
Trimipramine	1.14 (0.91–1.43)	1.31 (0.98–1.76)
Venlafaxine	0.97 (0.93–1.02)	1.10 (1.03–1.17)

*CI, Confidence interval; ROR, reporting odds ratio; SMQ, standardized MedDRA queries*.

### Preferred Term “Upper Gastrointestinal Haemorrhage”

In the analysis of ICSRs categorized as “suspected/interacting” regarding the PT “Upper gastrointestinal haemorrhage” (ICSRs related to antithrombotics included; no consideration of the competition bias) two signals were found: citalopram [ROR: 1.61 (1.15–2.25)] and escitalopram [ROR: 1.56 (1.06–2.29)]. Using ICSRs categorized as “suspected/interacting/concomitant,” eight signals were found: amitriptyline [ROR: 1.60 (95% CI, 1.31–1.96)], citalopram [ROR: 2.51 (2.14–2.93)], duloxetine [1.49 (1.23–1.81)], escitalopram [ROR: 2.23 (1.86–2.67)], mirtazapine [ROR: 3.05 (2.52–3.70)], paroxetine [ROR: 1.36 (1.11–1.66)], sertraline [ROR: 2.13 (1.84–2.45)], and trazodone [ROR: 2.29 (1.89–2.77)]. After removing reports associated with antithrombotics from the database (i.e., consideration of the competition bias) seven signals were found (using ICSRs categorized as “suspected/interacting/concomitant”); no new signals were detected; the previously found signals persisted with the exemption of paroxetine [ROR: 1.36 (1.11–1.66) vs. 1.13 (0.77–1.68)]. For the ROR values including the 95% CI for each substance for the PT “Upper gastrointestinal haemorrhage,” see Table 3.

**Table 3:** Reporting odds ratios and 95% CIs related to antidepressants and the PT “Upper gastrointestinal haemorrhage” with and without consideration of the competition bias ROR (95% CI).

**Substance**	**ROR without consideration of the competition bias**	**ROR in consideration of the competition bias**
Agomelatine	NA	NA
Amitriptyline	1.60 (1.31–1.96)	2.08 (1.49–2.90)
Bupropion	0.55 (0.40–0.76)	0.46 (0.25–0.86)
Citalopram	2.51 (2.14–2.93)	2.18 (1.59–2.98)
Clomipramine	0.22 (0.05–0.87)	0.66 (0.17–2.65)
Doxepin	1.49 (0.90–2.47)	1.31 (0.49–3.48)
Duloxetine	1.49 (1.23–1.81)	1.53 (1.08–2.17)
Escitalopram	2.23 (1.86–2.67)	2.02 (1.42–2.88)
Fluoxetine	1.11 (0.91–1.36)	1.21 (0.86–1.72)
Fluvoxamine	0.34 (0.11–1.07)	0.70 (0.17–2.78)
Hypericum perforatum	1.54 (0.50–4.78)	3.27 (0.82–13.08)
Imipramine	0.79 (0.36–1.76)	0.41 (0.06–2.92)
Maprotiline	NA	NA
Milnacipran	NA	NA
Mirtazapine	3.05 (2.52–3.70)	3.07 (2.14–4.39)
Moclobemide	NA	NA
Nortriptyline	1.23 (0.74–2.04)	1.40 (0.58–3.36)
Paroxetine	1.36 (1.11–1.66)	1.13 (0.77–1.68)
Reboxetine	0.59 (0.08–4.21)	NA
Sertraline	2.13 (1.84–2.45)	1.68 (1.25–2.25)
Tianeptine	NA	NA
Tranylcypromine	NA	NA
Trazodone	2.29 (1.89–2.77)	2.34 (1.64–3.33)
Trimipramine	0.71 (0.18–2.84)	2.26 (0.57–9.05)
Venlafaxine	1.15 (0.92–1.43)	1.27 (0.87–1.86)

*CI, confidence interval; NA, data not available (<3 records); PT, preferred term; ROR, reporting odds ratio; SMQ, standardized MedDRA queries*.

## Discussion

In the present study, we evaluated whether the causality assessment/categorization of a drug/event report as causally linked (“suspected/interacting”) vs. any type of association (“suspected/interacting/concomitant”) has an impact on signal detection using an example of the bleeding risk related to ADs. Furthermore, we analyzed the risk of different types of bleeding events and the influence of the competition bias by evaluating the effect of antithrombotics as competitors.

### Effects of Different Types of Association of Drug/Event Reports on Signal Detection

In a previous analysis, where we only analyzed causally linked drug/event-reports, we could not detect a significant number of signals, meaning that we were not able to demonstrate the well-known bleeding risk related to ADs (25). By contrast, in the present study, where we assessed ICSRs categorized as “suspected/interacting/concomitant,” we found several signals concerning bleeding in general, gastrointestinal bleeding, and upper gastrointestinal bleeding. These findings strengthen the evidence for an increased bleeding risk of ADs, particularly concerning the risk of bleedings of the gastrointestinal tract (7, 11, 26–28). Moreover, the results of our study suggest that the use of association categories implying a causal link between drug and event may impede the detection of signals in disproportionality analysis. This may be particularly true regarding ADRs that are not well-known, hard to clinically identify, or difficult to assign to a specific drug, namely, rare, or non-severe/clinically insignificant ADRs. It was previously suggested that treatment with ADs increases the bleeding risk only slightly (29). Moreover, it is possible that there is limited awareness regarding the increased risk of bleeding related to ADs among possible reports, e.g., physicians or patients. This may favor the reporting of bleeding events without indicating ADs as possibly causally linked and, in turn, affect the results of signal detection depending on the assigned association category. Our approach, where we analyzed all types of associations irrespective of the available information regarding causality, reduces specificity; however, it increases the sensitivity of the analysis. This approach may help to detect unknown, rare, or non-severe (clinically insignificant) ADRs.

### Competition Bias

Principally, by considering a possible competition bias caused by antithrombotic agents, there was no major impact of the competition bias on disproportionality analysis in the present study concerning ADs and bleeding; in other studies, however, the competition bias was found to be relevant for the sensitivity in signal detection (20–22): Pariente et al. unmasked signals for gastric and esophageal haemorrhages related to prednisone, rivastigmine, and isotretinoin regarding bleeding events by considering the competitive effects of antithrombotic agents (21); in a methodological work, the numbers of reports that are needed to detect a signal for bleeding events decreased after removing data related to competitive substances (22). In our study, however, after considering a possible competition bias caused by antithrombotic agents, we only found new signals for bleeding, in general, and for gastrointestinal bleeding related to the serotonin–norepinephrine reuptake inhibitors (SNRI) duloxetine and venlafaxine. In this regard, it has to be considered that antithrombotic agents are frequently associated with an increased risk of bleedings as their influence on hemostasis is desired, and bleeding can be a consequence of the pharmacological effect of the substance. Therefore, they are well-suited as competitors in the analyses of the bleeding events in pharmacovigilance databases (22). Several studies have consequently shown an even more increased risk of bleeding related to ADs combined with antithrombotic agents (6, 30, 31). In this regard, it has to be taken into account that in our study, we were indeed not able to “unmask” signals related to antidepressants after removal of ICSRs related to antithrombotics. However, signals related to ADs preponderantly persisted in all analyses, which speaks in favor of a relevant risk of bleeding related to ADs, even when administered without antithrombotics.

### Other Studies Regarding Abnormal Bleedings Related to Antidepressive Psychopharmacotherapy

Our results are in line with the current literature regarding antidepressants with serotonin reuptake inhibition and an associated increased risk of bleeding. The bleeding risk related to antidepressants has not yet been demonstrated on the basis of data from spontaneous reporting systems. However, there are several studies demonstrating an increased risk for haemorrhages related to ADs with different methods. In a population-based cohort study by Renoux et al., an association between an antidepressant's affinity for the serotonin transporter and the risk of intracranial haemorrhage was found; the risk was even further increased with oral anticoagulants as comedication (8). In a case-control study with more than 64,000 antidepressant users, there was a 2.6-fold increased bleeding risk for substances with a high degree of serotonin reuptake inhibition compared with a low one (32). A recent meta-analysis of 42 observational studies regarding the bleeding risk of SSRI showed an increase in the bleeding risk by at least 36% (33).

### Risk of Bleeding Related to Localization: Sensitivity vs. Specificity?

Taking into account the presumably particularly increased risk of bleeding related to ADs in the gastrointestinal tract [especially the upper gastrointestinal tract (11)], we also used more specific terms for the database query, namely, gastrointestinal haemorrhages and upper gastrointestinal haemorrhages. Although the case numbers were reduced in this subgroup analyses [total number of events “Haemorrhage” (SMQ): 990,119, “Gastrointestinal haemorrhage” (SMQ): 257,500, “Upper gastrointestinal haemorrhage” (PT): 10,908] several signals appeared, indicating an increased risk of bleeding events of the gastrointestinal tract related to ADs. These findings are in line with several studies about gastrointestinal bleeding events in association with antidepressant use. In an observational case-control study with 430,455 warfarin users, an increased risk of gastrointestinal bleeding was found for citalopram, fluoxetine, paroxetine, amitriptyline, and mirtazapine, which is in line with our findings except for fluoxetine (7). One retrospective cohort study with 317,824 elderly people enrolled found an association between antidepressants with serotonin reuptake inhibition and upper gastrointestinal bleeding (11). However, there are also studies suggesting no (34) or only a modest increase (26) in risk for upper gastrointestinal haemorrhage in association with ADs, which might explain why, in previous studies with a more sensitive approach, we were not able to find any signals.

## Limitations

One major limitation of the study is the common problem of underreporting in ADR databases (19, 35). In the special case of Ads and bleeding, this is of concern since bleeding events related to antidepressants could be frequently underreported because bleeding events like petechiae are often not severe and, thus, might not be reported (36). Another limitation of our study is the missing information on comedication besides the influence of antithrombotics, especially regarding NSAIDs that are also associated with an increased risk of bleeding, particularly regarding the upper gastrointestinal tract (37–40). In this analysis, out of the NSAIDs group, we only considered acetylsalicylic acid because it is also included in the antithrombotic agents group (ATC code B01), and the bleeding risk is considered a type A reaction. The influence of other NSAIDs (e.g., diclofenac or ibuprofen) that are associated with an increased risk of bleeding might also be relevant. Furthermore, since we have no data regarding actual prescription numbers, a quantitative comparison of the RORs in terms of the relative risk is not possible. A signal found with disproportionality analysis only show an association and does not allow deductions on absolute or relative risks.

## Conclusion

This is the first time the bleeding risk related to ADs could be demonstrated using pharmacovigilance data. In contrast to previous studies, drug/event reports were analyzed irrespective of the available information regarding causality. The results of the present study suggest that in disproportionality analysis, the restriction to association categories that imply a causal link between drug and event may impede the detection of signals, particularly regarding ADRs that are rare, unknown, non-severe (clinically insignificant), hard to recognize, or difficult to assign to a specific drug. Even after removal of all reports associated with antithrombotic agents, there were still signals related to ADs regarding an increased bleeding risk, indicating there is a relevant risk of bleeding related to ADs even when administered without antithrombotics.

## Data Availability Statement

Publicly available datasets were analyzed in this study. This data can be found here: https://www.who-umc.org/vigibase/vigibase/.

## Author Contributions

All authors contributed to the study conception and design. Material preparation, data collection, and analysis were performed by RZ and MG. The first draft of the manuscript was written by RZ and MG. All authors commented on previous versions of the manuscript and read and approved the final manuscript.

## Conflict of Interest

The authors declare that the research was conducted in the absence of any commercial or financial relationships that could be construed as a potential conflict of interest.

## Publisher's Note

All claims expressed in this article are solely those of the authors and do not necessarily represent those of their affiliated organizations, or those of the publisher, the editors and the reviewers. Any product that may be evaluated in this article, or claim that may be made by its manufacturer, is not guaranteed or endorsed by the publisher.

## References

[1] National Institute for Heath and Care Excellence. National Institute for Health and Care Excellence Depression in Adults: Treatment and Management. (2018). Available online at: https://www.nice.org.uk/guidance/gid-cgwave0725/documents/full-guideline-updated 35977056

[2] National Institute for Health and Care Excellence. Generalised Anxiety Disorder and Panic Disorder in Adults: Management. Natl Inst Heal Clin Excell. (2016). Available online at: https://www.nice.org.uk/guidance/cg113 31961629

[3] Forman-Hoffman V Middleton JC Feltner C Gaynes BN Weber RP Bann C. Psychological and pharmacological treatments for adults with posttraumatic stress disorder : a systematic review update. RTI Int North Carolina Chapel Hill Evidence-based Pract Cent. (2018) 616:72–5. 10.23970/AHRQEPCCER207 30204376

[4] American Psychological Association. Clinical Practice Guideline for the Treatment of Posttraumatic Stress Disorder (PTSD). Washington, DC APA: Guidel Dev Panel Treat Posttraumatic Stress Disord Adults (2017). p. 139.

[5] Czarny MJ Arthurs E Coffie DF Smith C Steele RJ Ziegelstein RC. Prevalence of antidepressant prescription or use in patients with acute coronary syndrome: a systematic review. PLoS ONE. (2011) 6:e27671. 10.1371/journal.pone.0027671 22132126 PMC3222644

[6] Schalekamp T Klungel OH Souverein PC de Boer A. Increased bleeding risk with concurrent use of selective serotonin reuptake inhibitors and coumarins. Arch Intern Med. (2008) 168:180–5. 10.1001/archinternmed.2007.32 18227365

[7] Schelleman H Brensinger CM Bilker WB Hennessy S. Antidepressant-warfarin interaction and associated gastrointestinal bleeding risk in a case-control study. PLoS ONE. (2011) 6:e21447. 10.1371/journal.pone.0021447 21731754 PMC3123326

[8] Renoux C Vahey S Dell'Aniello S Boivin J-F. Association of selective serotonin reuptake inhibitors with the risk for spontaneous intracranial hemorrhage. JAMA Neurol. (2017) 74:173. 10.1001/jamaneurol.2016.4529 27918771

[9] Douros A Ades M Renoux C. Risk of intracranial hemorrhage associated with the use of antidepressants inhibiting serotonin reuptake: a systematic review. CNS Drugs. (2018) 32:321–34. 10.1007/s40263-018-0507-7 29536379

[10] Roose SP Rutherford BR. Selective serotonin reuptake inhibitors and operative bleeding risk: a review of the literature. J Clin Psychopharmacol. (2016) 36:704–9. 10.1097/JCP.0000000000000575 27684291 PMC5093043

[11] van Walraven C Mamdani MM Wells PS Williams JI. Inhibition of serotonin reuptake by antidepressants and upper gastrointestinal bleeding in elderly patients: retrospective cohort study. BMJ. (2001) 323:655–8. 10.1136/bmj.323.7314.655 11566827 PMC55923

[12] de Abajo FJ García-Rodríguez LA. Risk of upper gastrointestinal tract bleeding associated with selective serotonin reuptake inhibitors and venlafaxine therapy: interactions with nonsteroidal anti-inflammatory drugs and effect of acid-suppressing agents. Arch Gen Psychiatry. (2008) 65:795–803. 10.1001/archpsyc.65.7.795 18606952

[13] de Abajo FJ Rodríguez LA Montero D. Association between selective serotonin reuptake inhibitors and upper gastrointestinal bleeding : population based case-control study. BMJ. (1999) 319:1106–9. 10.1136/bmj.319.7217.1106 10531103 PMC28262

[14] Dalton SO Johansen C Mellemkjaer L Norgard B Sorensen HT Olsen JH. Use of selective serotonin reuptake inhibitors and risk of upper gastrointestinal bleeding: a population-based cohort study. Arch Intern Med. (2003) 163:59–64. 10.1001/archinte.163.1.59 12523917

[15] Gahr M Zeiss R Lang D Connemann BJ Hiemke C Freudenmann RW. Risk of bleeding related to selective and non-selective serotonergic antidepressants: a case/non-case approach using data from two pharmacovigilance databases. Pharmacopsychiatry. (2015) 48:19–24. 10.1055/s-0034-1394398 25376976

[16] Maschino F Hurault-Delarue C Chebbane L Fabry V Montastruc JL Bagheri H. Bleeding adverse drug reactions (ADRs) in patients exposed to antiplatelet plus serotonin reuptake inhibitor drugs: analysis of the French Spontaneous Reporting Database for a controversial ADR. Eur J Clin Pharmacol. (2012) 68:1557–60. 10.1007/s00228-012-1268-8 22527341

[17] de Boissieu P Kanagaratnam L Abou Taam M Roux MP Drame M Trenque T. Notoriety bias in a database of spontaneous reports: the example of osteonecrosis of the jaw under bisphosphonate therapy in the French national pharmacovigilance database. Pharmacoepidemiol Drug Saf. (2014) 23:989–92. 10.1002/pds.3622 24737486

[18] Williams D Feely J. Underreporting of adverse drug reactions: attitudes of Irish doctors. Ir J Med Sci. (1999) 168:257–61. 10.1007/BF02944353 10624366

[19] Martin RM Kapoor KV Wilton LV Mann RD. Underreporting of suspected adverse drug reactions to newly marketed (“black triangle”) drugs in general practice: observational study. BMJ. (1998) 317:119–20. 10.1136/bmj.317.7151.119 9657787 PMC28603

[20] Salvo F Leborgne F Thiessard F Moore N Bégaud B Pariente A. A potential event-competition bias in safety signal detection: results from a spontaneous reporting research database in France. Drug Saf. (2013) 36:565–72. 10.1007/s40264-013-0063-5 23673817

[21] Pariente A Avillach P Salvo F Thiessard F Miremont-Salamé G Fourrier-Reglat A. Effect of competition bias in safety signal generation. Drug Saf. (2012) 35:855–64. 10.1007/BF03261981 22967190

[22] Pariente A Didailler M Avillach P Miremont-Salamé G Fourrier-Reglat A Haramburu F. A potential competition bias in the detection of safety signals from spontaneous reporting databases. Pharmacoepidemiol Drug Saf. (2010) 19:1166–71. 10.1002/pds.2022 20848561

[23] UMC. VigiBase: Signalling Harm and Pointing to Safer Use. Available online at: https://www.who-umc.org/vigibase/vigibase/vigibase-signalling-harm-and-pointing-to-safer-use/ (accessed April 16, 2020).

[24] International Conference on Harmonisation of Technical Requirements For Registration of Pharmaceuticals for Human Use. Maintenance of the ICH Guideline on Clinical Safety Data Management E2B(R2) (2001).

[25] Zeiss R Hiemke C Schönfeldt C Connemann BJ Gahr M. Risk of bleeding associated with antidepressant drugs: the competitive impact of antithrombotics in quantitative signal detection. Drugs Real World Outcomes. (2021). 10.1007/s40801-021-00260-9. [Epub ahead of print]. PMC8605951 34117617

[26] Anglin R Yuan Y Moayyedi P Tse F Armstrong D Leontiadis GI. Risk of upper gastrointestinal bleeding with selective serotonin reuptake inhibitors with or without concurrent nonsteroidal anti-inflammatory use: a systematic review and meta-analysis. Am J Gastroenterol. (2014) 109:811–9. 10.1038/ajg.2014.82 24777151

[27] Wang YP Chen YT Tsai CF Li SY Luo JC Wang SJ. Short-term use of serotonin reuptake inhibitors and risk of upper gastrointenstinal bleeding. Am J Psychiatry. (2014) 171:54–61. 10.1176/appi.ajp.2013.12111467 24030313

[28] Jiang H-Y Chen H-Z Hu X-J Yu Z-H Yang W Deng M. Use of selective serotonin reuptake inhibitors and risk of upper gastrointestinal bleeding: a systematic review and meta-analysis. Clin Gastroenterol Hepatol. (2015) 13:42–50. 10.1016/j.cgh.2014.06.021 24993365

[29] Andrade C Sandarsh S Chethan KB Nagesh KS. Serotonin reuptake inhibitor antidepressants and abnormal bleeding: a review for clinicians and a reconsideration of mechanisms. J Clin Psychiatry. (2010) 71:1565–75. 10.4088/JCP.09r05786blu 21190637

[30] Hackam DG Mrkobrada M. Selective serotonin reuptake inhibitors and brain hemorrhage: a meta-analysis. Neurology. (2012) 79:1862–5. 10.1212/WNL.0b013e318271f848 23077009

[31] Labos C Dasgupta K Nedjar H Turecki G Rahme E. Risk of bleeding associated with combined use of selective serotonin reuptake inhibitors and antiplatelet therapy following acute myocardial infarction. CMAJ. (2011) 183:1835–43. 10.1503/cmaj.100912 21948719 PMC3216455

[32] Meijer WEE Heerdink ER Nolen WA Herings RMC Leufkens HGM Egberts ACG. Association of risk of abnormal bleeding with degree of serotonin reuptake inhibition by antidepressants. Arch Intern Med. (2004) 164:2367. 10.1001/archinte.164.21.2367 15557417

[33] Laporte S Chapelle C Caillet P Beyens M-N Bellet F Delavenne X. Bleeding risk under selective serotonin reuptake inhibitor (SSRI) antidepressants: a meta-analysis of observational studies. Pharmacol Res. (2017) 118:19–32. 10.1016/j.phrs.2016.08.017 27521835

[34] Carvajal A Ortega S Del Olmo L Vidal X Aguirre C Ruiz B. Selective serotonin reuptake inhibitors and gastrointestinal bleeding: a case-control study. PLoS ONE. (2011) 6:e19819. 10.1371/journal.pone.0019819 21625637 PMC3097219

[35] Hazell L Shakir SA. Under-reporting of adverse drug reactions : a systematic review. Drug Saf. (2006) 29:385–96. 10.2165/00002018-200629050-00003 16689555

[36] Lopez-Gonzalez E Herdeiro MT Figueiras A. Determinants of under-reporting of adverse drug reactions. Drug Saf. (2009) 32:19–31. 10.2165/00002018-200932010-00002 19132802

[37] de Jong JC van den Berg PB Tobi H de Jong-van den Berg LT. Combinded use of SSRIs and NSAIDs increases the risk of gastrointestinal adverse effects. Br J Clin Pharmacol. (2003) 55:591–5. 10.1046/j.0306-5251.2002.01770.x 12814454 PMC1884264

[38] Zullino DF Khazaal Y. Increased risk of gastrointestinal adverse effects under SSRI/NSAID combination may be due to pharmacokinetic interactions. Br J Clin Pharmacol. (2005) 59:118–9. 10.1111/j.1365-2125.2005.2212_1.x 15606451 PMC1884968

[39] Shin J-YY Park M-JJ Lee SH Choi S-HH Kim M-HH Choi N-KK. Risk of intracranial haemorrhage in antidepressant users with concurrent use of non-steroidal anti-inflammatory drugs: nationwide propensity score matched study. BMJ. (2015) 351:h3517. 10.1136/bmj.h3517 26173947 PMC4501372

[40] Loke YK Trivedi AN Singh S. Meta-analysis: gastrointestinal bleeding due to interaction between selective serotonin uptake inhibitors and non-steroidal anti-inflammatory drugs. Aliment Pharmacol Ther. (2008) 27:31–40. 10.1111/j.1365-2036.2007.03541.x 17919277

