# Corrigendum: Risk of Bleeding Associated With Antidepressants: Impact of Causality Assessment and Competition Bias on Signal Detection

**DOI:** 10.3389/fpsyt.2021.813879

**Published:** 2021-12-21

**Authors:** René Zeiss, Bernhard J. Connemann, Carlos Schönfeldt-Lecuona, Maximilian Gahr

**Affiliations:** Department of Psychiatry and Psychotherapy III, University of Ulm, Ulm, Germany

**Keywords:** antidepressants, serotonin transporter, bleeding risk, pharmacovigilance, competition bias

In the original article, the abstract was incorrect. Unfortunately, an older version of the abstract was included in the final proof. In the following, the correct abstract referring to the above mentioned article is presented.

Corrections have been made to the **Introduction, Results, Methods**, and **Conclusion**:

**Introduction:** It has not yet been possible to demonstrate the well-established increased bleeding risk related to antidepressants (ADs) with methods of pharmacovigilance as disproportionality analysis. As bleeding events related to ADs often occur under comedication with antithrombotics, ADs might not be considered causative of, but merely “linked” with the bleeding event. Therefore, we hypothesized that causality assessment of bleeding events related to ADs and the competitive impact of antithrombotics are factors contributing to the mentioned previous non-findings.

**Methods:** We performed a case/non-case study based on data from VigiBase™ and calculated reporting odds ratios (RORs) for 25 ADs. We used individual case safety reports (ICSRs) that were differently categorized in the database regarding the type of association between drug and event. Furthermore, we investigated the competitive impact of antithrombotics by comparing RORs calculated with and without ICSRs related to antithrombotics.

**Results:** Analysis of ICSRs that were categorized as causally associated with ADs resulted in detection of only 2 signals (citalopram and escitalopram; upper gastrointestinal bleeding). Analysis of ICSRs irrespective of the type of association resulted in detection of 8 signals (regarding bleeding in general, gastrointestinal bleeding and upper gastrointestinal bleeding). In our analysis, consideration of ICSRs associated with antithrombotics as competitive substances did not have significant impact on signal detection.

**Conclusion:** Categorization of the type of association between drug and event may affect quantitative signal detection toward reduced sensitivity. Causality assessment seems to significantly impact signal detection, probably particularly in rare, unknown, or clinically unremarkable adverse drug reactions. ADs appear to increase the bleeding risk considerably, even independent of antithrombotic comedication.

The authors apologize for this error and state that this does not change the scientific conclusions of the article in any way. The original article has been updated.

## Author Contributions

All authors listed have made a substantial, direct, and intellectual contribution to the work and approved it for publication.

## Publisher's Note

All claims expressed in this article are solely those of the authors and do not necessarily represent those of their affiliated organizations, or those of the publisher, the editors and the reviewers. Any product that may be evaluated in this article, or claim that may be made by its manufacturer, is not guaranteed or endorsed by the publisher.

