# Supplementary material for: Risk of Bleeding Associated With Antidepressants: Impact of Causality Assessment and Competition Bias on Signal Detection
Source: Front Psychiatry. 2021 Oct 21;12:727687. doi: 10.3389/fpsyt.2021.727687 (PMC8566746; doi:10.3389/fpsyt.2021.727687)
Supplement: Supplementary file 1 [file Data_Sheet_1.docx]

Supplementary Material

| Table S1: Reporting odds ratios for antidepressants and the SMQ “Haemorrhages” (narrow scope) for category “suspected/interacting” and “suspected/interacting/concomitant”  ROR (95 % CI) | | |
| --- | --- | --- |
| Substance | **ROR “suspected/interacting”** | **ROR “suspected/interacting/concomitant”** |
| Agomelatine | 0.32 (0.25-0.41) | 0.54 (0.45-0.65) |
| *Amitriptyline* | *0.35 (0.32-0.38)* | *1.09 (1.06-1.12)* |
| Bupropion | 0.32 (0.31-0.34) | 0.79 (0.77-0.81) |
| *Citalopram* | *0.86 (0.82-0.9)* | *1.41 (1.38-1.45)* |
| Clomipramine | 0.41 (0.36-0.47) | 0.59 (0.54-0.65) |
| Doxepin | 0.34 (0.29-0.41) | 0.99 (0.92-1.06) |
| Duloxetine | 0.59 (0.56-0.62) | 0.97 (0.95-1.00) |
| *Escitalopram* | *0.88 (0.84-0.93)* | *1.35 (1.32-1.39)* |
| *Fluoxetine* | *0.74 (0.71-0.77)* | *1.03 (1.01-1.05)* |
| Fluvoxamine | 0.49 (0.43-0.55) | 0.62 (0.57-0.68) |
| *Hypericum perforatum* | *1.04 (0.84-1.28)* | *1.26 (1.10-1.45)* |
| Imipramine | 0.31 (0.26-0.38) | 0.80 (0.73-0.87) |
| Maprotiline | 0.32 (0.25-0.41) | 0.52 (0.44-0.62) |
| Milnacipran | 0.43 (0.35-0.53) | 0.77 (0.68-0.87) |
| Mirtazapine | 0.31 (0.28-0.34) | 1.04 (1.00-1.08) |
| Moclobemide | 0.23 (0.17-0.3) | 0.37 (0.30-0.45) |
| Nortriptyline | 0.35 (0.29-0.41) | 1.06 (1.00-1.12) |
| *Paroxetine* | *0.63 (0.61-0.66)* | *1.05 (1.02-1.07)* |
| Reboxetine | 0.32 (0.24-0.44) | 0.40 (0.31-0.51) |
| *Sertraline* | *0.67 (0.65-0.7)* | *1.22 (1.20-1.25)* |
| Tianeptine | 0.31 (0.21-0.44) | 1.10 (0.95-1.26) |
| Tranylcypromine | 0.77 (0.61-0.97) | 0.72 (0.58-0.88) |
| *Trazodone* | *0.28 (0.25-0.32)* | *1.28 (1.25-1-32)* |
| Trimipramine | 0.42 (0.31-0.55) | 0.86 (0.75-0.98) |
| Venlafaxine | 0.59 (0.56-0.61) | 0.97 (0.95-1.00) |
| Abbreviations/legend: CI=Confidence Interval, ROR=Reporting Odds Ratio, SMQ=Standardised MedDRA Queries, Substances that demonstrated a signal after switching the association category from „suspected/interacting“ to „suspected/interacting/concomitant“ are highlighted in italic letters | | |

| Table S2: Reporting odds ratios and 95% CIs related to antidepressants and the SMQ “Gastrointestinal haemorrhage” in the categories “suspected/interacting” vs. “suspected/interacting/concomitant” | | |
| --- | --- | --- |
| Substance | **ROR “suspected/interacting”** | **ROR “suspected/interacting/concomitant”** |
| Agomelatine | 0.24 (0.13-0.43) | 0.42 (0.28-0.62) |
| Amitriptyline | *0.25 (0.21-0.31)* | *1.43 (1.37-1.50)* |
| Bupropion | 0.24 (0.21-0.27) | 0.69 (0.65-0.73) |
| Citalopram | *0.96 (0.88-1.05)* | *1.67 (1.60-1.74)* |
| Clomipramine | 0.13 (0.08-0.21) | 0.48 (0.40-0.58) |
| Doxepin | *0.18 (0.11-0.29)* | *1.28 (1.14-1.43)* |
| Duloxetine | 0.62 (0.57-0.67) | 1.01 (0.96-1.06) |
| Escitalopram | *0.85 (0.76-0.94)* | *1.37 (1.30-1.43)* |
| Fluoxetine | 0.5 (0.46-0.55) | 0.96 (0.92-1.00) |
| Fluvoxamine | 0.39 (0.29-0.5) | 0.63 (0.53-0.75) |
| Hypericum perforatum | 0.54 (0.31-0.93) | 1.02 (0.77-1.36) |
| Imipramine | 0.11 (0.06-0.21) | 0.95 (0.82-1.10) |
| Maprotiline | 0.1 (0.04-0.23) | 0.45 (0.32-0.63) |
| Milnacipran | 0.32 (0.2-0.5) | 0.46 (0.34-0.62) |
| Mirtazapine | *0.21 (0.17-0.26)* | *1.41 (1.33-1.49)* |
| Moclobemide | 0.16 (0.08-0.31) | 0.46 (0.33-0.65) |
| Nortriptyline | 0.17 (0.1-0.27) | 1.05 (0.93-1.17) |
| Paroxetine | *0.46 (0.42-0.5)* | *1.10 (1.05-1.15)* |
| Reboxetine | 0.22 (0.1-0.46) | 0.37 (0.22-0.62) |
| Sertraline | *0.54 (0.5-0.59)* | *1.31 (1.26-1.36)* |
| Tianeptine | 0.12 (0.04-0.39) | 1.01 (0.77-1.34) |
| Tranylcypromine | 0.2 (0.08-0.49) | 0.27 (0.14-0.52) |
| Trazodone | *0.2 (0.15-0.26)* | *1.56 (1.49-1.64)* |
| Trimipramine | 0.17 (0.07-0.4) | 1.14 (0.91-1.43) |
| Venlafaxine | 0.5 (0.45-0.55) | 0.97 (0.93-1.02) |
| Abbreviations/legend: CI=Confidence Interval, ROR=Reporting Odds Ratio, SMQ=Standardised MedDRA Queries; Substances that demonstrated a signal after switching the association category from „suspected/interacting“ to „suspected/interacting/concomitant“ are highlighted in italic letters | | |

| Table S3: Reporting odds ratios for antidepressants and the SMQ PT “Upper gastrointestinal haemorrhage” for category “suspected/interacting” and “suspected/interacting/concomitant”  ROR (95 % CI) | | |
| --- | --- | --- |
| Substance | **ROR “suspected/interacting”** | **ROR “suspected/interacting/concomitant”** |
| Agomelatine | NA | NA |
| Amitriptyline | *0.06 (0.01-0.44)* | *1.60 (1.31-1.96)* |
| Bupropion | 0.03 (0-0.19) | 0.55 (0.40-0.76) |
| Citalopram | 1.61 (1.15-2.25) | 2.51 (2.14-2.93) |
| Clomipramine | NA | 0.22 (0.05-0.87) |
| Doxepin | NA | 1.49 (0.90-2.47) |
| *Duloxetine* | *0.39 (0.23-0.66)* | *1.49 (1.23-1.81)* |
| Escitalopram | 1.56 (1.06-2.29) | 2.23 (1.86-2.67) |
| Fluoxetine | 0.52 (0.35-0.78) | 1.11 (0.91-1.36) |
| Fluvoxamine | NA | 0.34 (0.11-1.07) |
| Hypericum perforatum | 1.98 (0.49-7.93) | 1.54 (0.50-4.78) |
| Imipramine | NA | 0.79 (0.36-1.76) |
| Maprotiline | NA | NA |
| Milnacipran | NA | NA |
| *Mirtazapine* | *0.28 (0.11-0.75)* | *3.05 (2.52-3.70)* |
| Moclobemide | NA | NA |
| Nortriptyline | 0.25 (0.04-1.78) | 1.23 (0.74-2.04) |
| *Paroxetine* | *0.83 (0.59-1.16)* | *1.36 (1.11-1.66)* |
| Reboxetine | 0.74 (0.1-5.28) | 0.59 (0.08-4.21) |
| *Sertraline* | *0.88 (0.63-1.22)* | *2.13 (1.84-2.45)* |
| Tianeptine | NA | NA |
| Tranylcypromine | NA | NA |
| *Trazodone* | *0.09 (0.01-0.65)* | *2.29 (1.89-2.77)* |
| Trimipramine | NA | 0.71 (0.18-2.84) |
| Venlafaxine | 0.52 (0.33-0.82) | 1.15 (0.92-1.43) |
| Abbreviations/legend: CI=Confidence Interval, ROR=Reporting Odds Ratio, SMQ=Standardised MedDRA Queries, Substances that demonstrated a signal after switching the association category from „suspected/interacting“ to „suspected/interacting/concomitant“ are highlighted in italic letters | | |
